# In Vitro Co-Culture Model of Primary Human Osteoblasts and Osteocytes in Collagen Gels

**DOI:** 10.3390/ijms20081998

**Published:** 2019-04-23

**Authors:** Jasmin Skottke, Michael Gelinsky, Anne Bernhardt

**Affiliations:** Centre for Translational Bone, Joint and Soft Tissue Research, Medical Faculty and University Hospital, Technische Universität, 01307 Dresden, Germany; jasmin.skottke@tu-dresden.de (J.S.); michael.gelinsky@tu-dresden.de (M.G.)

**Keywords:** osteoblast, osteocyte, three-dimensional (3D) co-culture, osteocalcin, collagen gel

## Abstract

Background: Osteocytes are the key regulator cells in bone tissue, affecting activity of both osteoblasts and osteoclasts. Current in vitro studies on osteocyte-osteoblast interaction are invariably performed with rodent cells, mostly murine cell lines, which diminishes the clinical relevance of the data. Objective: The objective of the present study was to establish an in vitro co-culture system of osteoblasts and osteocytes, which is based solely on human primary cells. Methods: Three different approaches for the generation of human primary osteocytes were compared: direct isolation of osteocytes from bone tissue by multistep digestion, long-time differentiation of human pre-osteoblasts embedded in collagen gels, and short time differentiation of mature human osteoblasts in collagen gels. Co-cultivation of mature osteoblasts with osteocytes, derived from the three different approaches was performed in a transwell system, with osteocytes, embedded in collagen gels at the apical side and osteoblasts on the basal side of a porous membrane, which allowed the separate gene expression analysis for osteocytes and osteoblasts. Fluorescence microscopic imaging and gene expression analysis were performed separately for osteocytes and osteoblasts. Results: All examined approaches provided cells with typical osteocytic morphology, which expressed osteocyte markers E11, osteocalcin, phosphate regulating endopeptidase homolog, X-linked (PHEX), matrix extracellular phosphoglycoprotein (MEPE), sclerostin, and receptor activator of NF-κB Ligand (RANKL). Expression of osteocyte markers was not significantly changed in the presence of osteoblasts. In contrast, osteocalcin gene expression of osteoblasts was significantly upregulated in all examined co-cultures with differentiated osteocytes. Alkaline phosphatase (ALPL), bone sialoprotein II (BSPII), and RANKL expression of osteoblasts was not significantly changed in the co-culture. Conclusion: Interaction of osteoblasts and osteocytes can be monitored in an in vitro model, comprising solely primary human cells.

## 1. Introduction

Osteocytes are the major cell type in the bone and account for up to 95% of bone cells [1]. They are located in mineralized bone and are in contact with osteoblasts on the bone surface via gap junctions [2]. The poor accessibility of osteocytes and their difficult cultivation have only recently made it possible to develop a suitable isolation method [3] and in vitro environment [4]. It has been supposed, that osteocytes orchestrate bone homeostasis by regulating bone-forming osteoblasts and bone-resorbing osteoclasts. Interactions between osteoblasts and osteocytes have already been demonstrated: mature osteocytes express sclerostin and Dkk1, which have a negative effect on the Wnt/β-catenin pathway. This pathway plays an important role in the regulation of bone mass by osteoblasts and its inhibition suppresses the activity of osteoblasts [5,6,7]. This relationship is confirmed by sclerostin (SOST) knockout mice and Dkk mutant mice, which showed a high bone mass with increased bone formation [8,9].

In vitro co-culture of osteocytes and osteoblasts are an additional approach to study the interaction of these cell types. Some studies on in vitro co-cultivation were already published. Taylor and co-workers cultivated osteocytes and osteoblasts on different sides of a transwell insert membrane [10], however 2D cultivation of osteocytes does not imitate the situation in vivo, where osteocytes are embedded into the 3D bone matrix. Other groups embedded osteocytes in collagen gels and seeded osteoblasts on top of this construct [11,12]. The missing spatial separation of the two cell types and the potential of osteoblasts to migrate into collagen gels can cause cross-contamination of the RNA between osteocytes and osteoblasts, which makes separate gene expression analysis impossible. In addition, all published co-culture studies used cell lines for osteoblasts and osteocytes (e.g., MLO-Y4 and MC3T3-E1) although immortalized cell lines do not fully resemble the behavior of primary cells due to phenotypic and genetic differences [3,13]. Therefore, an in vitro co-culture model of osteoblasts and osteocytes, comprising solely human primary cells would be highly advantageous. Such in vitro co-cultures can be used for further investigations of osteocyte-osteoblast interaction. Additionally, the effect of growth factors or drugs on the bone cell interaction can be studied.

The main aim of this study was to develop a functional co-culture with suitable conditions for osteocytes and osteoblasts and to identify interactions between the two cell types. Therefore, osteocytes were cultivated in a 3D collagen matrix to ensure a natural bone environment. Three different approaches to obtain human primary osteocytes were compared, to find the optimal approach for co-cultivation: (i) direct isolation of primary human osteocytes from bone tissue by sequential digestion and demineralization, (ii) long-term differentiation of pre-osteoblasts in collagen gels, and (iii) short time differentiation of mature osteoblasts in collagen gels. The three types of osteocyte cultures were embedded in collagen gels, which were inserted into commercially available transwell inserts. Mature osteoblasts were seeded onto the basal side of the porous transwell insert membrane, enabling the separation of osteocytes and osteoblasts for analysis. In all co-culture experiments, primary osteoblasts and osteocytes derived from the same donor to imitate the conditions in human bone.

## 2. Results

### 2.1. Comparison of Isolated Osteocytes with Osteocytes, Differentiated from Pre-Osteoblasts and Mature Osteoblasts

**Figure 1:**
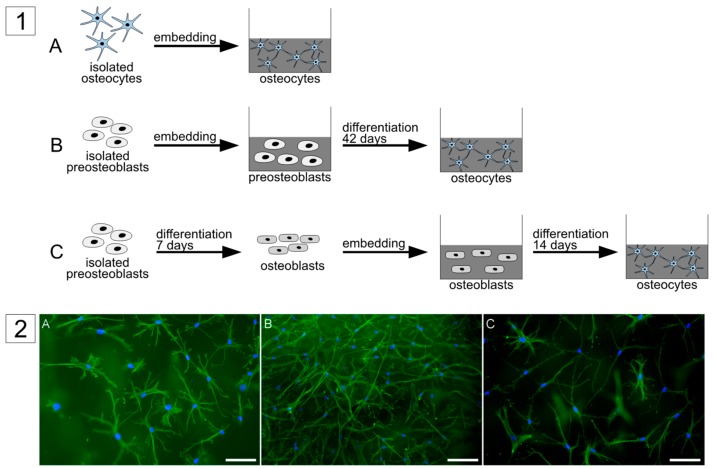
(**1**) Schematic diagram of different approaches to obtain primary human osteocytes in collagen gels: (**A**) freshly isolated human primary osteocytes were embedded for 2 days in collagen gel, (**B**) human pre-osteoblasts were embedded in collagen gel and cultivated for 6 weeks, (**C**) human pre-osteoblasts were differentiated using dexamethasone, ascorbic acid 2-phosphate, and β-glycerophosphate for 7 days on tissue culture polystyrene (TCPS) before embedding them in collagen gels for another 14 days. (**2**) Fluorescence images of osteocytes in collagen gels after staining the cytoskeleton (Alexa 488, green) and nuclei (DAPI (4′,6-diamidin-2-phenylindol, Invitrogen), blue). Scale bar represents 100 µm.

**Figure 2:**
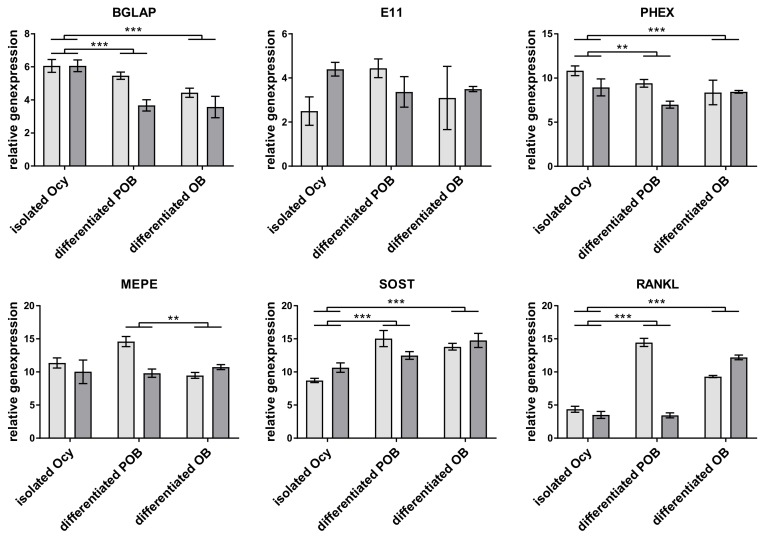
Relative expression of osteocyte-specific genes from isolated and in vitro differentiated osteocytes. Diagrams show the ΔCt value in relation to the expression of glyceraldehyde-3-phosphate dehydrogenase (GAPDH). Data from two different cell donors are presented in one diagram (*n* = 3 replicates/donor): mean ± sd. Significant differences were defined as * *p* < 0.05, ** *p* < 0.01, and *** *p* < 0.001 (two-way ANOVA).

Three different approaches were used in this study to obtain primary human osteocytes (Figure 1). The first approach was to directly isolate the cells from human bone by a multistep digestion protocol as described before [3,4]. Cells were embedded into collagen gels and analyzed after two days of cultivation. The second approach was to embed primary pre-osteoblasts, which were isolated from human bone by single step collagenase digestion, into collagen gels and to cultivate them for 6 weeks, similar to a previous study [14]. The third approach was to pre-differentiate pre-osteoblasts for 7 days on tissue culture polystyrene (TCPS), using dexamethasone, ascorbic acid 2 phosphate, and β-glycerophosphate, before embedding them into a collagen gel and further cultivating them for 14 days. For all these approaches the quality of osteocytes was checked by morphological evaluation and gene expression analysis. After fluorescence staining of cytoskeleton and nuclei of the gel-embedded cells, the occurrence of dendritic excesses, originating from one cells into different directions was considered. Further characteristic of osteocytes is the expression of E11, PHEX, MEPE, sclerostin, RANKL, and osteocalcin. All three approaches were successful in generating cells with osteocyte-like morphology: long dendritic extensions originating from one cell body in different directions (Figure 1). Gene expression analysis revealed expression of osteocyte-specific markers in all approaches (Figure 2). There were slight differences between the groups: isolated osteocytes expressed more SOST and RANKL and less PHEX and Bone gamma carboxyglutamate protein, osteocalcin (BGLAP) than both in vitro differentiated osteocytes. Differentiated pre-osteoblasts expressed less MEPE than in vitro differentiated osteoblasts. Apart from these differences, all approaches are suitable to generate osteocytes and were therefore applied for co-culture studies.

### 2.2. Co-Culture Studies

**Figure 3:**
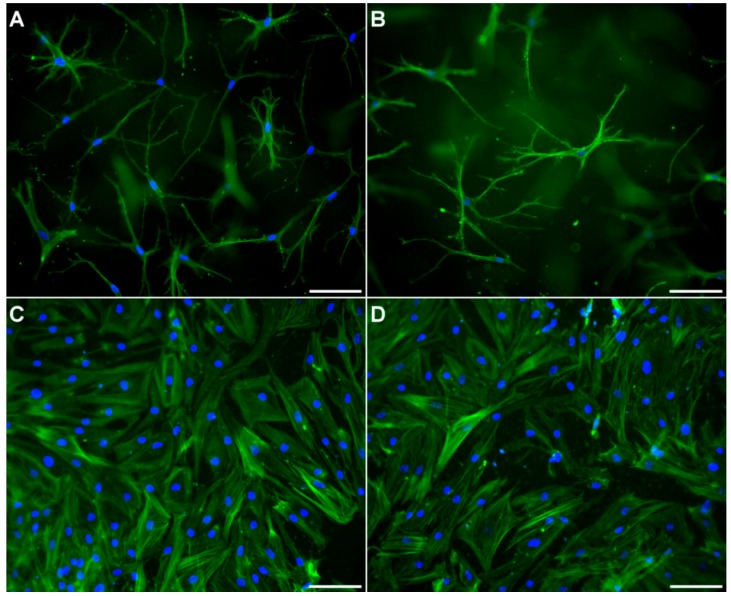
Osteocytes (**A**,**B**) and osteoblasts (**C**,**D**) in single (**A**,**C**) and co-culture (**B**,**D**). Osteocytes were obtained by in vitro differentiation from mature osteoblasts in collagen gels in a transwell insert. Osteoblasts were differentiated from pre-osteoblasts and added on the outer surface of the insert membrane. Fluorescence images of osteocytes after staining the cytoskeleton (Alexa 488, green) and nuclei (DAPI, blue). Scale bar represents 100 µm.

All co-culture studies were performed in transwell inserts, with osteoblasts, pre-differentiated from human pre-osteoblasts, on the basal side of the membrane and osteocytes, freshly isolated or differentiated from osteoblasts embedded in collagen gel. Osteocytes showed dendritic morphology, both in single- and co-cultures of all three approaches. Osteoblasts showed their typical cobblestone-like morphology, both in single- and co-culture of all three approaches. Representative images of osteocytes and osteoblasts in single- and co-culture are exemplarily shown in Figure 3.

#### 2.2.1. Cocultures with Freshly Isolated Osteocytes

**Figure 4:**
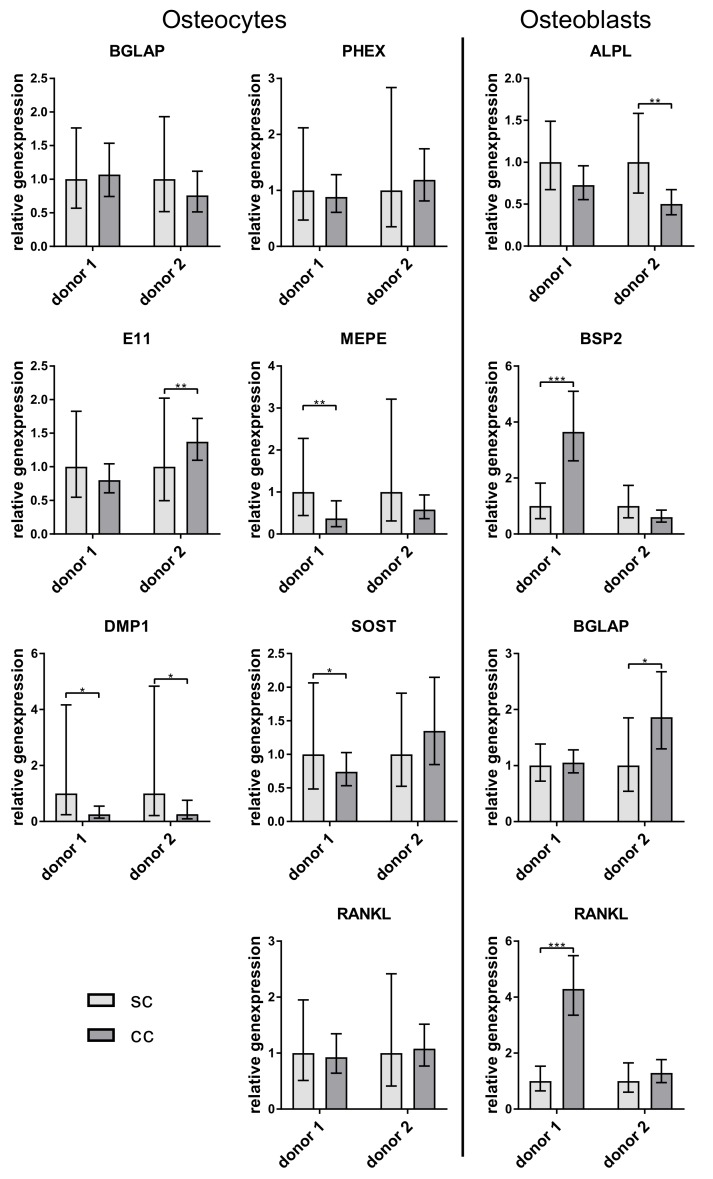
Relative expression of osteocyte and osteoblast markers from single- (sc) and co-cultivated (cc) osteocytes and osteoblasts. Osteocytes were isolated directly from bone (compare Figure 1A). Data from two different cell donors are presented in one diagram (*n* = 3 replicates/donor): mean ± upper/lower limit. Significant differences to the single culture were defined as* *p* < 0.05, ** *p* < 0.01, and *** *p* < 0.001.

Gene expression of osteocytes and osteoblasts did not show huge differences between single and coculture. Only DMP1 expression was significantly lower in the osteocytes from the co-culture (Figure 4).

#### 2.2.2. Coculture with in Vitro Differentiated Osteocytes from Pre-Osteoblasts

**Figure 5:**
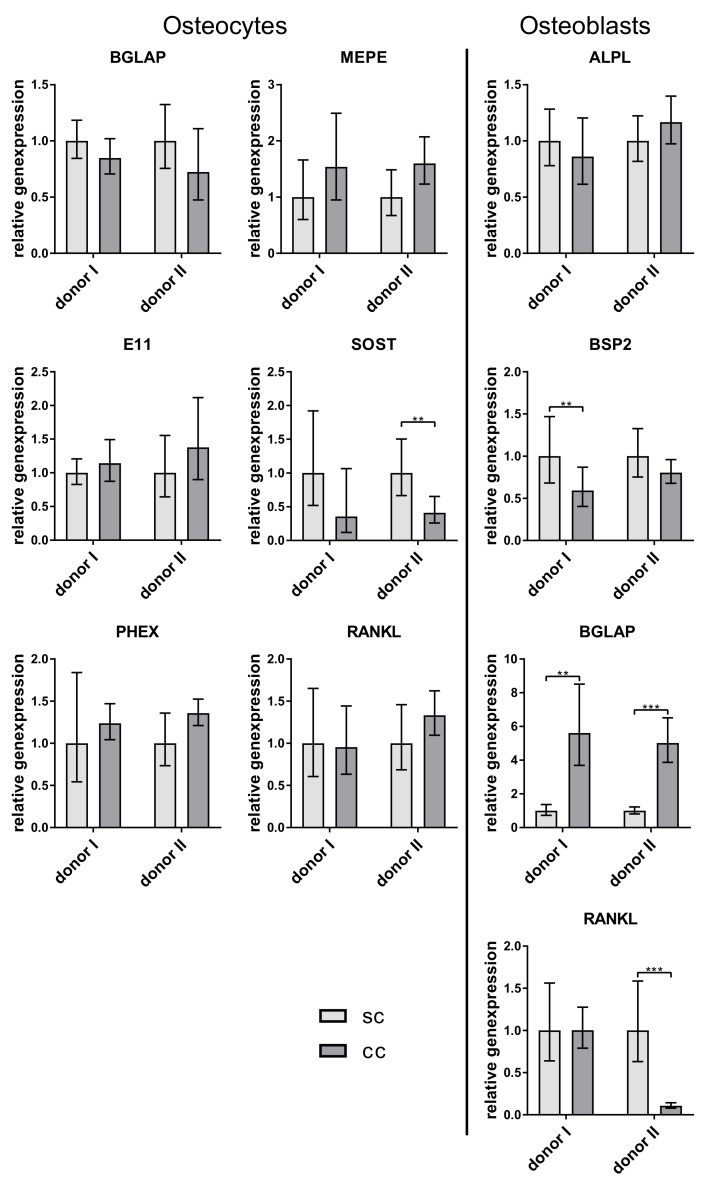
Relative expression of osteocyte and osteoblast markers from single- (sc) and co-cultivated (cc) osteocytes and osteoblasts. Osteocytes were generated by 6 week pre-cultivation of osteoblasts in collagen gels (compare Figure 1B). Data from two different cell donors are presented in one diagram (*n* = 3 replicates/donor): mean ± upper/lower limit. Significant differences to the single culture were defined as ** *p* < 0.01 and *** *p* < 0.001.

No repeatable difference in gene expression of osteocytic marker and RANKL was detected between single- and co-cultured in vitro differentiated osteocytes. In contrast, osteoblasts expressed significantly more BGLAP when co-cultured with the osteocytes (Figure 5).

#### 2.2.3. Coculture with in Vitro Differentiated Osteocytes from Mature Osteoblasts

**Figure 6:**
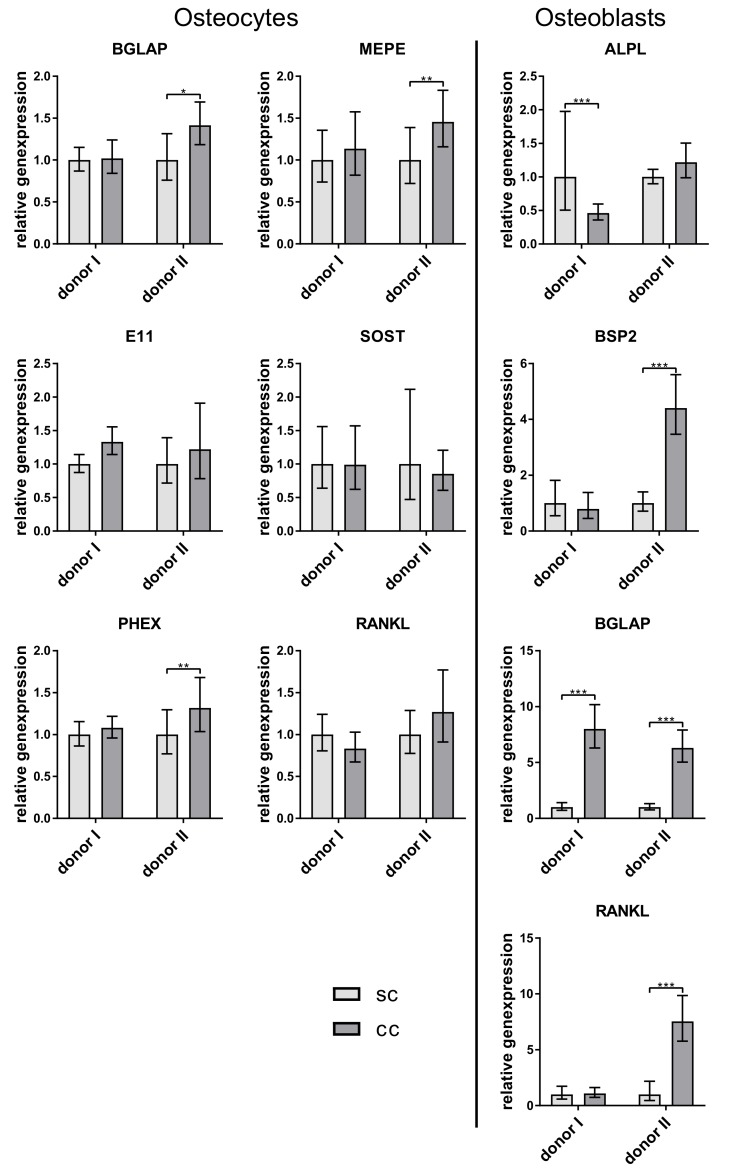
Relative expression of osteocyte and osteoblast markers from single- (sc) and co-cultivated (cc) osteocytes and osteoblasts. Osteocytes were prepared by 2-week differentiation of pre-differentiated osteoblasts in collagen gels (compare Figure 1C). Data from two different cell donors are presented in one diagram (*n* = 3 replicates/donor): mean ± upper/lower limit. Significant differences to the single culture were defined as * *p* < 0.05, ** *p* < 0.01, and *** *p* < 0.001.

In this culture, the osteocytes again showed no change in their gene expression and osteoblasts expressed more BGLAP in co-culture (Figure 6).

## 3. Discussion

In this paper, a co-culture of primary osteocytes and osteoblasts was established, to investigate the interaction of these bone cell types. Some challenges have prevented the development of such co-cultures so far: primary osteocytes are difficult to culture, because they need a 3D collagen matrix in vitro to form dendritic extensions and maintain their osteocytic phenotype [4]. Adding osteoblasts onto the surface of collagen gels results in a migration process of the osteoblasts from the surface into the gel and simultaneous differentiation to osteocytes [12,14]. Therefore, a spatial separation from the osteoblasts to the collagen gel is necessary. Additionally, osteocyte cultivation requires a medium with low fetal calf serum (FCS) [3,15], while the common medium for osteoblasts contains 10% FCS. Thus, a medium supporting the differentiation of both cell types had to be found. It has been shown that the cultivation of osteoblasts under low FCS concentrations promotes the osteogenic phenotype [16]. Therefore, medium containing 2% FCS was suitable for both cell types in the co-culture.

Current in vitro studies on osteocyte-osteoblast interaction were invariably performed with rodent cells, and the present study is the first to establish an in vitro co-culture model comprising solely primary human osteocytes and osteoblasts. One of the aims of this study was to identify suitable conditions for the generation of human primary osteocytes. Therefore, we compared three approaches: (i) direct isolation of osteocytes from bone tissue by multistep digestion, (ii) long-time differentiation of human pre-osteoblasts embedded in collagen gels, and (iii) short-time differentiation of mature human osteoblasts in collagen gels. With all approaches it was possible to obtain differentiated dendritic osteocytes expressing osteocytic marker including SOST, which cannot be observed in the commonly used MYO-Y4 osteocytes [17].

To decide on an approach suitable for co-culture experiments, the time factor and the gained cell amount play an important role. Since the multi-step digestion of bone to receive primary osteocytes is very time consuming and only a few cells are obtained, the first method is not very efficient. The second approach needs a long period of six weeks differentiation from pre-osteoblasts to osteocytes. During the first period of differentiation, the cells proliferate, resulting in a high final osteocyte number in the gels. Those high osteocyte numbers bear the risk of gel shrinking [4]. Furthermore, the osteocyte morphology might be more difficult to evaluate in gels with a high cell density. The last approach with differentiation of mature osteoblasts just needs three weeks including pre-differentiation of the osteoblasts and is therefore suggested as the easiest, fastest, and most efficient approach to obtain primary osteocytes.

Although there are already a few approaches for co-cultures of osteocytes and osteoblasts [10,11,12], differences between single and co-cultures of osteocytes and osteoblasts have not been examined. Our study showed a stable expression of osteocyte markers both in single and co-culture. All osteocytes expressed sclerostin, a protein that was shown to inhibit the osteogenic differentiation and the function of the osteoblasts [6]. However, in the co-culture experiments of our study, no downregulation of the examined osteoblast markers ALPL, BSPII, and RANKL was observed. Probably the amount of sclerostin which was synthesized by the osteocytes in vitro was too low to have an effect on the osteoblasts. Further studies should involve the quantification of SOST not only on gene but also on protein level.

RANKL is expressed by osteocytes and osteoblasts and promotes osteoclastogenesis [18]. It has been shown for murine cells, that osteocytes express even more RANKL than osteoblasts [19]. In our experiments, there was no clearly higher RANKL expression in osteocytes. We detected both higher and lower RANKL expression in osteocytes compared to osteoblasts of the same experiment (data not shown). However, freshly isolated osteocytes showed the highest RANKL expression (Figure 2). A very interesting observation of our co-culture study was that osteoblasts expressed significantly more osteocalcin when cultivated with osteocytes. This is the first time that a stimulation of osteoblasts by osteocytes, which were not mechanically treated before, was shown in vitro. Regulatory elements in the promoter of the osteocalcin gene were already characterized, indicating a strong transcriptional regulation of this gene. This regulation is mainly controlled by transcription factors like Runx2, AP-1 related proteins, or homeobox proteins [20].

Several in vitro studies indicated that Runx2 (Cbfa1/AML3) is a positive regulator of osteocalcin expression at an early stage of osteoblast maturation [21,22]. While at a late stage of maturation, the overexpression of Runx2 in osteoblasts failed to upregulate osteocalcin expression. This indicates that some other factors are required for the regulation of osteocalcin expression at the later stages [22]. Since we used mature osteoblasts for co-cultures, the increase in osteocalcin expression may not be caused by a regulation of the Runx2 transcription factor. AP-1 is a transcription factor that binds DNA by forming a heterodimer composed of proteins belonging to the c-Fos and c-Jun families (c-Jun, JunB and JunD, c-Fos, FosB, Fra-1, and Fra-2) [23]. Grigoriadis et al. demonstrated that osteoblastic cells are principal targets for c-Fos [24]. However, they observed a reduction of osteocalcin expression in cell lines with high c-fos expression. Mason et al. proved that normal and mechanically stimulated osteocytes in the cortical bone of ulnae of rats constitutively express c-Fos and c-Jun [25]. These data suggest that c-Fos and c-Jun, expressed by osteocytes, might be involved in the gene regulation of osteocalcin in osteoblasts. Homebox proteins (Msx1, Msx2, Dlx5, and Dlx6) play a role in osteoblast differentiation and osteocalcin expression, too. Yet, an effect of dlx3 and dlx5 on the osteocalcin gene expression has been demonstrated [23].

A regulation of these transcription factors by osteocytes is possible by their release of growth factors like BMP2, TGFβ, IGF-1, PGE2, FGF-2, and others. Their effect has mainly been studied on the Runx2transcription factor, but BMP2 also promotes Dlx5 expression in osteoblasts, which activates osteocalcin expression [23].

The function of osteocalcin in the co-culture could not yet be investigated, but various studies demonstrated that osteocalcin is a regulator of mineralization by binding calcium ions [26].

In contrast to the effects of osteocytes on osteoblast osteocalcin expression, the present study did not show any significant effects of osteoblasts on osteocyte gene expression. This observation may suggest, that possible interactive effects of osteoblasts on osteocytes are negligible, however, it is still possible that those effects exist in a narrow zone of osteocytes, which is in close proximity to the osteoblast layer.

In summary, it can be concluded that osteocytes stimulate osteocalcin expression in osteoblasts even without mechanical stimulation or external factors. However, this stimulation could be further enhanced by such additional factors, which can be further investigated. The regulation of the osteocalcin gene by osteocytes is a very complex mechanism, involving many different factors. Because osteocalcin has skeletal and metabolic functions [26], further research for a better understanding of this regulation is necessary, to offer new therapeutic strategies against bone diseases and metabolic disorders. The co-culture established is this paper is suitable for such studies.

## 4. Materials and Methods

### 4.1. Isolation of Primary Human Osteocytes and Pre-Osteoblasts

Osteocytes and pre-osteoblasts were isolated from human femoral heads of osteoarthritic patients undergoing total hip replacement at the University Hospital Carl Gustav Carus Dresden (Germany) after informed consent (approval by the ethics commission of TU Dresden, EK 3030814, 8. August. 2014). For osteocyte isolation bone material of two donors was used (donor 1: female 67 years, donor 2: male 77 years). For pre-osteoblast isolation bone material of the above mentioned two donors, and additionally, from another two donors was used (donor I: female 56 years, donor II: female 75 years). Osteocyte isolation was performed based on a protocol of Prideaux and co-workers [3] with some modifications (Bernhardt et al. Biomedical Engineering, in press). Spongious bone fragments (1–2 mm) were repeatedly digested (collagenase II treatment) and demineralized (EDTA treatment). Resting steps of the particles in between the treatments increased the yield of osteocytes. Isolated primary osteocytes after at least six digestion steps were cultivated on collagen-coated TCPS for 2 days with α-MEM, 2% fetal calf serum (FCS), 2 mM L-glutamine, 100 U/mL penicillin and 100 µg/mL streptomycin, before embedding the cells into collagen gels. Pre-osteoblasts were isolated from spongious bone fragments (1–2 mm) after two collagenase digestions. Cells were expanded in α-MEM, 15% FCS, 2 mM L-glutamine, 100 U/mL penicillin and 100 µg/mL streptomycin, before use in the different experiments.

### 4.2. Differentiation of Pre-Osteoblasts

Pre-osteoblasts were differentiated in vitro for 7 days in α-MEM, 10% FCS, 10 mM β-glycerophosphate, 50 µM ascorbic acid 2-phosphate, 10^−7^ M dexamethasone, 2 mM L-glutamine, 100 U/mL penicillin, and 100 µg/mL streptomycin to get mature osteoblasts.

### 4.3. Preparation of Collagen Gels

Collagen gels were prepared by mixing 9 parts of acidic collagen solution (3 mg/mL rat collagen in 0.1 M acetic acid, Amedrix, Esslingen, Germany) with 1 part of 10× HBSS and neutralizing with 1 M NaOH. For embedding of osteocytes, pre-osteoblasts or osteoblasts the respective cell suspension was added to the neutralized collagen at a concentration of 1 × 10^5^ cells/mL. Cell laden collagen solution (0.5 mL) was added to 12-well transwell inserts with 0.4 µm pore size.

### 4.4. Co-Culture

Transwell inserts, filled with cell-laden collagen gels (osteocytes, directly isolated from bone or differentiated from osteoblasts, according to the respective approach) were inverted to add 1 × 10^5^ pretreated osteoblasts (always from the same donor as the osteocytes) onto the basal side of the membrane, and incubated for 4 h at 37 °C to allow cellular adhesion. Afterwards the inserts were reverted and the constructs were cultivated in α-MEM, 2% FCS, 50 µM ascorbic acid 2-phosphate, 10 mM β-glycerophosphate, 2 mM L-glutamine, 100 U/mL penicillin and 100 µg/mL streptomycin. Additionally, single cultures of osteocytes, embedded in collagen gels in transwell inserts and single cultures of osteoblasts on the basal side of transwell inserts, which were filled with cell-free collagen gels were performed. Three replicates were used per culture and for every replicate, two samples were pooled.

### 4.5. Fluorescence Microscopy

Osteocyte-containing gels were removed from the transwell inserts and osteoblast-seeded membranes were cut out of the insert with a scalpel. Gels and membranes were fixed separately with a 4% solution of neutral buffered formaldehyde for 1 h at RT. After permeabilization with 0.1% Triton X-100 in PBS for 5 min and 6 washes with PBS, samples were blocked using 3% bovine serum albumin in PBS for 30 min. The samples were further incubated with a solution of 20 ng/mL DAPI (4′,6-diamidin-2-phenylindol, Invitrogen) and 25 µL/mL AlexaFluor 488^®^ phalloidin (Invitrogen, part of Thermo Fisher Scientific, Waltham, MA, USA) overnight at 4 °C protected from light. After removal of the staining solution, the samples were finally washed with PBS and imaged with a Keyence BZ 9000 fluorescence microscope.

### 4.6. RNA Isolation and cDNA Synthesis

Cell-seeded collagen gels (six 0.5 mL-gels per experimental group) were removed from the transwell inserts and incubated with collagenase II solution (3 mg/mL collagenase II, 235 U/mg, Biochrom, Berlin, Germany in α-MEM, 10% FCS, 2 mM L-glutamine, 100 U/mL penicillin and 100 µg/mL streptomycin, 3 mM CaCl_2_) for 1 h at 37 °C. The digests were transferred to 15 mL tubes, washed with PBS and centrifuged. The pellets were washed with PBS and RNA was isolated from these pellets as well as from the osteoblast-seeded transwell membranes using a commercially available kit (peqGOLD MicroSpin Total RNA Kit, Peqlab, Erlangen, Germany). cDNA was generated using the High-Capacity cDNA Reverse Transcription Kit (Applied Biosystems, part of Thermo Fisher Scientific) according to manufacturer’s instructions.

### 4.7. PCR

PCR reactions were set up using the TaqMan Fast Advanced Master Mix (Thermo Fisher) and TaqMan Gene Expression Assays for glyceraldehyde-3-phosphate dehydrogenase (GAPDH), bone gamma-carboxyglutamate protein (osteocalcin, BGLAP), podoplanin (E11/g38; PDPN), phosphate regulating endopeptidase homolog, X-linked (PHEX), matrix extracellular phosphoglycoprotein (OPF 45, MEPE), receptor activator of NF-κB Ligand (RANKL, TNFSF11), dentin matrix protein 1 (DMP-1), sclerostin (SOST), alkaline phosphatase (ALPL), and bone sialoprotein II (BSP II) (Applied Biosystems, part of Thermo Fisher Scientific) according to manufacturer’s instructions.

PCR was run with an Applied Biosystems^®^ 7500 fast Real-Time PCR system (Applied Biosystems, part of Thermo Fisher Scientific). Relative gene expression (fold-change) was calculated using the 2^−ΔΔCt^ method and normalized to GAPDH expression. For the comparison of different approaches to generate primary human osteocytes, the ΔCT values were directly compared.

Statistical differences of different groups were calculated at the level of ΔCT values using one-way or two-way ANOVA followed by Post hoc Tukey test to determine multiple comparisons (Origin 9.1, OriginLab).

## Abbreviations

2D/3D	Two-/Three-dimensional
ALPL	Alkaline phosphatase liver/bone/kidney
BGLAP	Bone gamma carboxyglutamate protein, osteocalcin
BSP2	Bone Sialoprotein 2
DMP1	Dentin matrix acidic phosphoprotein 1
E11	Podoplanin
FCS	Fetal calf serum
GAPDH	Glyceraldehyde 3-phosphate dehydrogenase
MEPE	Matrix Extracellular Phosphoglycoprotein
PHEX	Phosphate-regulating neutral endopeptidase, X-linked
RANKL	Receptor activator of nuclear factor κB ligand
RT-qPCR	Reverse transcribed polymerase chain reaction
SOST	Sclerostin
TCPS	Tissue Culture Polystyrene
